# Research on Synthetic Aperture Radar Processing for the Spaceborne Sliding Spotlight Mode

**DOI:** 10.3390/s18020455

**Published:** 2018-02-03

**Authors:** Shijian Shen, Xin Nie, Xinggan Zhang

**Affiliations:** 1 School of Electronic Science and Engineering, Nanjing University, Nanjing 210023, China; shenshj@163.com; 2 Nanjing Research Institute of Electronics Technology, Nanjing 210039, China; 13913863864@139.com

**Keywords:** synthetic aperture radar (SAR), sliding spotlight mode, CBP algorithm, PFA algorithm, Gaofen-3

## Abstract

Gaofen-3 (GF-3) is China’ first C-band multi-polarization synthetic aperture radar (SAR) satellite, which also provides the sliding spotlight mode for the first time. Sliding-spotlight mode is a novel mode to realize imaging with not only high resolution, but also wide swath. Several key technologies for sliding spotlight mode in spaceborne SAR with high resolution are investigated in this paper, mainly including the imaging parameters, the methods of velocity estimation and ambiguity elimination, and the imaging algorithms. Based on the chosen Convolution BackProjection (CBP) and PFA (Polar Format Algorithm) imaging algorithms, a fast implementation method of CBP and a modified PFA method suitable for sliding spotlight mode are proposed, and the processing flows are derived in detail. Finally, the algorithms are validated by simulations and measured data.

## 1. Introduction

Gaofen-3 (GF-3), launched on 10 August 2016, is China’s first C-band multi-polarization synthetic aperture radar (SAR) satellite, mainly used in the fields of ocean surveillance, land observation, disaster reduction, and water conservation [1]. With 12 imaging modes, GF-3 not only covers the traditional stripmap and scan mode, but also provides the sliding spotlight mode for the first time [2]. The resolution of sliding spotlight mode in GF-3 is 1 meter, which is the highest resolution in a C-band multi-polarized SAR satellite in the world.

Stripmap mode and spotlight mode are two of the most common modes of operation in SAR [3]. Stripmap mode can provide images of large areas continuously, but the azimuth resolution cannot be arbitrarily increased; spotlight mode is achieved by controlling the scanning speed of the antenna so that it can fix on a certain ground area to improve the coherent integration time (CPI), so the azimuth resolution can be higher, but the imaging range is limited by the antenna beam width. In GF-3, sliding spotlight mode is a novel SAR mode, which increases the azimuth resolution by controlling the speed of the antenna irradiation to the area on the ground, and its imaging area is larger than the spotlight mode and its resolution can be higher than that of stripmap mode with the same size antenna [4]. Sliding spotlight mode is a good compromise between high-resolution and large-area imaging. At present, foreign advanced SAR systems, such as PAMIR and TerraSAR-X, all have adopted this imaging mode [5].

In this paper, the key techniques of spaceborne sliding spotlight SAR such as satellite imaging geometrical parameters and ambiguity elimination are analyzed, and two SAR algorithms—Convolution BackProjection (CBP) and Polar Format Algorithm (PFA)—are studied.

The back projection algorithm comes from the field of computer-aided tomography (CAT) [6] and is a point-by-point image reconstruction algorithm which has unique advantages [7,8]: no restrictions on the size of the imaging scene, available partial magnification of any part of the scene of interest, and no frequency domain interpolation is needed to ensure image quality. The biggest drawback of CBP algorithm, however, is the large amount of computation. The fast CBP algorithm has two directions: one is to divide the sub-apertures, and the other is to divide sub-images. The methods improve the efficiency based on the space or time sampling redundancy in the CBP algorithm. Sub-aperture method [9,10] refers to the technique whereby the whole aperture is divided into several smaller sub-apertures, each corresponding to a narrow range of bandwidth, which means that the sampling frequency in the azimuth domain can be lower to reduce the required interpolation so as to reduce the computation. The image of each sub-aperture is of low resolution, and the high resolution image is finally obtained by their coherent superposition. In sub-image methods [11,12], the entire imaging is divided into many small sub-images to reduce the spatial bandwidth in both range and azimuth domain. Correspondingly, the signal sampling rate can be reduced in two dimensions in order to improve the computation efficiency. In this paper, the fast realization method of CBP based on Quadtree recursive sub-image segmentation is studied, which is realized by sub-image interception, fast filtering and down-sampling to reduce the computation.

PFA is a classical spotlight SAR imaging algorithm [13,14], which is simple and efficient and also very suitable for sliding spotlight mode. Because of the assumption of plane wave front, the residual phase error will cause defocus in PFA to limit the size of the effective imaging scene. Because of the larger range of the imaging scene in sliding spotlight mode, the range of the small effective scene is not sufficient to meet the actual requirements. A modified PFA algorithm based on sub-aperture processing for wide scene with high resolution was proposed by Dorry [15], and has been improved in [16], but the overlap rate between two adjacent sub-apertures is very high, and the process is very complex. In this paper, based on the CBP algorithm of Quadtree recursive sub-image segmentation, it is further extended to the PFA algorithm, which greatly improves the effective imaging scene size of PFA. Finally, the proposed algorithms are verified by simulation and measured data.

## 2. Model of Spaceborne Sliding Spotlight SAR

### 2.1. Geometric Model

The sliding spotlight mode is a compromise between the stripmap mode and the spotlight mode [3]. In this mode, the radar slows down the moving speed of the antenna by controlling the beam direction, and increases the coherent integration time to obtain a higher azimuth resolution than possible with the traditional stripmap mode. At the same time, because the antenna beam still has a certain moving speed on the ground, a larger imaging area can be obtained than with the spotlight mode.

**Figure 1:**
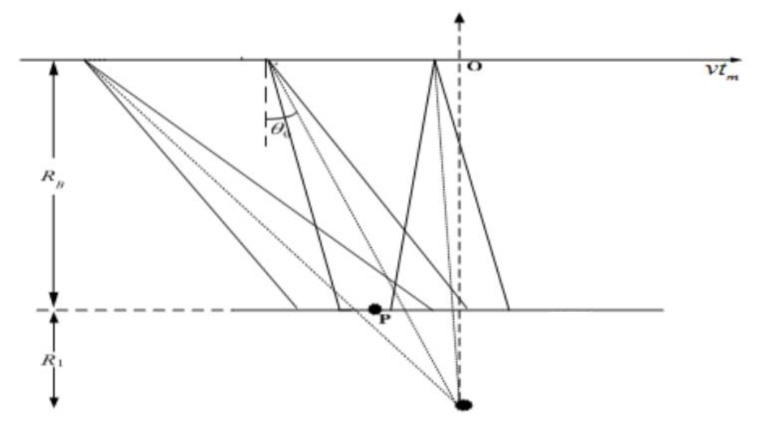
Basic model of sliding spotlight SAR.

The basic model of sliding spotlight SAR is shown in Figure 1. The horizontal axis is the slow time *t_m_*, and the satellite moves at a constant speed *v* along the axis. *R_B_* is the vertical distance between the satellite and the point *P*. During the flight, the antenna beam center always directs to a point on the ground (shown as the black dot in Figure 1, the antenna always points to a certain imaginary point in the spotlight mode, and to a hypothetical point underground in sliding spotlight mode). The origin *O* of the axis is the time at which the satellite is located directly at the point of the imaginary point, that means the distance from the imaginary point to the satellite is shortest during the flight time. According to the squint angle, the azimuth coordinates of the satellite can be obtained in each pulse.

Typical orbit parameters for spaceborne SAR simulation is shown in Table 1.

### 2.2. The Acquisition of Parameters in Spaceborne SAR

**Figure 2:**
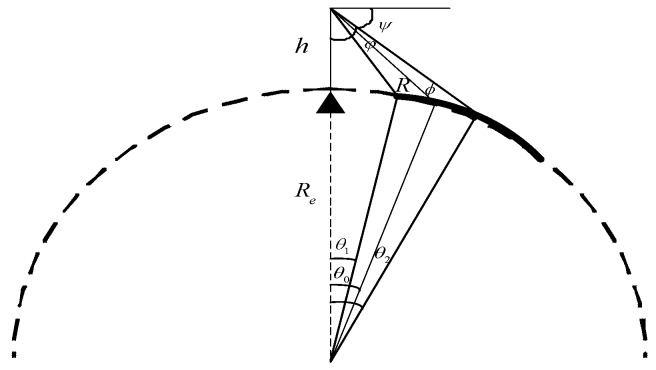
Geometric model of spaceborne sliding spotlight SAR.

The geometric pattern of spaceborne SAR is shown in Figure 2. Suppose the radius of the Earth is *R_e_*, the height of the satellite orbit from the ground is *h*, and if the center *φ*_0_ is known, the other main parameters can be calculated according to the following formulas [3]:

Corresponding center angle:

(1) $$\phi_{0}=\mathrm{asin}[(R_{e}+h)/R_{e}\cdot \sin\varphi_{0}]$$

Center slanting distance:

(2) $$R_{s0}=(R_{e}+h)\cos\varphi_{0}-\sqrt{R_{e}^{2}\cos^{2}\varphi_{0}-(h^{2}+2hR_{e})\sin^{2}\varphi_{0}}$$

The orbit of the satellite is approximately an elliptical eccentricity, and in general, the orbit can be treated as a circle. If the orbit is a circumference, the relationship between the orbital period *P* and the orbit radius *R_s_* is as follows:

(3) $$P^{2}=\frac{4\pi^{2}{R_{s}}^{3}}{\mu_{e}}$$

*μ_e_* = 3.986 × 10^14^ m^3^/s^2^ is the Earth gravitational constant. Correspondingly, the angular velocity of the satellite is:

(4) $$\omega_{s}=\frac{2\pi }{P}=\sqrt{\frac{\mu_{e}}{{R_{s}}^{3}}}$$

The satellite inertia speed is:

(5) $$V_{s}=R_{s}\omega_{s}=\sqrt{\frac{\mu_{e}}{R_{s}}}$$

*R_s_ = R_e_ + h*, *R_e_* is the radius of the Earth, and *h* is the height of satellite orbit. Then the velocity of the satellite can be obatined.

The slanting distance between satellite and targets is the most important parameter in SAR processing. This distance varies with azimuth and time. Assuming that the flight path is a partial line, the Earth is locally flat and not rotating. The distance between the satellite and the target point is *R*(*η*), which is given by the following hyperbolic equation:

(6) $$R\left(\eta \right)=\sqrt{{R_{0}}^{2}+{\left(V_{r}\eta \right)}^{2}}$$

In this hypothetical case, hyperbolic equation is also suitable for spaceborne situations, but *V* is not the physical speed, but the virtual speed chosen to make the real distance equation conform to the hyperbolic model.

**Figure 3:**
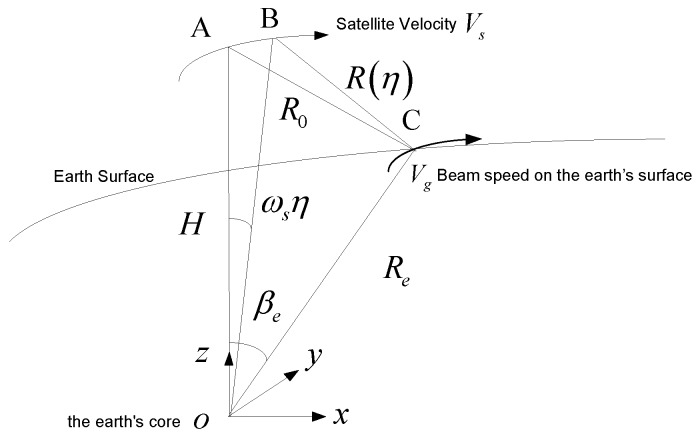
The geometric relationship, indicating the speed of satellite and beam coverage area.

As shown in Figure 3, the distance between the target and the satellite at time *η* is:

(7) $$R\left(\eta \right)=\sqrt{{\left(R_{e}\sin\beta_{e}\right)}^{2}+{\left(H\sin\omega_{s}\eta \right)}^{2}+{\left(H\cos\omega_{s}\eta R_{e}-R_{e}\cos\beta_{e}\right)}^{2}}$$

In the case of small angle, the distance in the upper form can be expressed as:

(8) $$\begin{array}{ll} R\left(\eta \right) & \approx \sqrt{H^{2}+R_{e}^{2}-2HR_{e}\cos\beta_{e}+(H\omega_{s})(R_{e}\omega_{s}\cos\beta_{e})\eta^{2}}\\ & =\sqrt{R_{0}^{2}+(H\omega_{s})(R_{e}\omega_{s}\cos\beta_{e})\eta^{2}} \end{array}$$

(9) $$R_{0}=\sqrt{H^{2}+R_{e}^{2}-2HR_{e}\cos\beta_{e}}$$

According to the hypothesis of the local circular orbit, *Hω_s_* is the orbital velocity of the satellite *V_s_*, while *R_e_ω_s_*cos*β_e_* is the velocity of the beam coverage rate, that is the value of the ground speed *V_g_* based on the assumption that the earth is locally spherical near the point *C*, so *V_g_* is parallel to *V_s_*.

The final result is as follows:

(10) $$R\left(\eta \right)=\sqrt{{R_{0}}^{2}+V_{s}V_{g}\eta^{2}}$$

The equivalent velocity can be obtained by:

(11) $$V_{r}\approx \sqrt{V_{g}V_{s}}$$

## 3. Ambiguity Elimination in Sliding Spotlight SAR

In spaceborne SAR, the selection of the PRF is usually only about 1.2 times the Doppler bandwidth produced by the antenna illumination range [17]. The relationship between the Doppler bandwidth required in the SAR and the resolution is as follows:

(12) $$B_{\min}=\frac{V_{r}}{\rho_{a}}$$

In the spaceborne sliding spotlight SAR, since the PRF selection is limited by many conditions, the PRF is usually only slightly larger than the instantaneous Doppler bandwidth, and is much smaller than the entire signal Doppler bandwidth. The Doppler ambiguity phenomenon exists in the echos, so it is necessary to study the solution of eliminating ambiguity in sliding spotlight SAR.

**Figure 4:**
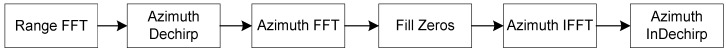
Flow chart of ambiguity elimination.

This section uses the dechirp operation to resolve the Doppler ambiguity [18]. The reference function of the SPECAN operation is selected to be related to the frequency of the distance. The difference in the SPENCAN operation results in different operations of phase compensation and interpolation. The specific algorithm flow is shown in Figure 4.

The biggest difference between sliding spotlight SAR and spotlight SAR is that the imaging area of sliding spotlight SAR is larger than the radiation area of the antenna. Therefore, if we do the dechirp operation at the center of scene, the Doppler bandwidth is still greater than the pulse repetition frequency, so the azimuth data need to be divided into several sub-apertures, each sub-aperture can have a certain overlap [19].

In the analysis of algorithm, we only consider the oblique plane imaging. In sliding spotlight mode, the echo signal can be written as follows:

(13) $$S\left(\omega ,u_{mohu}\right)=\sum \sigma \left(x_{n},r_{n}\right)\exp\left(-j\frac{2\left(\omega +\omega_{c}\right)}{c}\sqrt{{\left(x_{n}-u_{mo}\right)}^{2}+{\left(R_{c}+r_{n}\right)}^{2}}\right).$$

In the above formula, *R_c_* is the satellite’s distance to the center of the scene, *u_mo_* is the position of the satellite at each moment.

Use the following signal forms to dechirp data:

(14) $$S_{de}\left(\omega ,u\right)=\exp\left(j\frac{2\left(\omega +\omega_{c}\right)}{c}\sqrt{{\left(u_{a}\right)}^{2}+{\left(R_{c}\right)}^{2}}\right).$$

The echo after dechirp can be expressed as:

(15) $$S_{1}\left(\omega ,n\right)=\sum_{i=1}^{N}\sigma \left(x_{n},r_{n}\right)\exp\left(-j\frac{2\left(\omega +\omega_{c}\right)}{c}\left(\frac{R_{c}r_{n}-n\cdot \Delta u_{a}x_{n}}{\sqrt{n^{2}\cdot {\left(\Delta u_{a}\right)}^{2}+R_{c}^{2}}}+\xi \left(n\cdot \Delta u_{a},x_{n},r_{n}\right)\right)\right).$$

Δ*u_mohu_ = V_r_/PRF*, *ξ*(*n*·Δ*u_mohu_*, *x_n_*, *r_n_*) is residual error term based on the plane wave assumption. The Doppler bandwidth of the signal is only determined by the scene-induced Doppler bandwidth. That is to say, if the scene-induced Doppler bandwidth in the sub-aperture is more than the pulse repetition rate, then we can use the interpolation method to increase PRF. In the interpolation process, we can perform the Fourier transform on the above equation, and then perform the inverse Fourier transform after the zero-padding, which is equivalent to interpolating the above equation. The number of zeroes is determined by the number of ambiguity. After interpolation, the signal can be expressed as:

(16) $$S_{1}\left(\omega ,i\right)=\sum_{i=1}^{N}\sigma \left(x_{n},r_{n}\right)\exp\left(-j\frac{2\left(\omega +\omega_{c}\right)}{c}\left(\frac{R_{c}r_{n}-i\cdot \Delta ux_{n}}{\sqrt{i^{2}\cdot {\left(\Delta u\right)}^{2}+R_{c}^{2}}}+\xi \left(i\cdot \Delta u,x_{n},r_{n}\right)\right)\right).$$

In order to recover the echo signal without ambiguity, inverse dechirp processing also needs to be performed. In this case, the compensation vector for inverse dechirp processing is:

(17) $$S_{Ide}\left(\omega ,i\right)=\exp\left(j\frac{2\left(\omega +\omega_{c}\right)}{c}\sqrt{{\left(i\Delta u\right)}^{2}+{\left(R_{c}\right)}^{2}}\right).$$

After inverse dechirp processing, the echo can be expressed as follows:

(18) $$S\left(\omega ,i\right)=\sum_{i=1}^{N}\sigma \left(x_{n},r_{n}\right)\exp\left(-j\frac{2\left(\omega +\omega_{c}\right)}{c}\sqrt{{\left(x_{n}-i\cdot \Delta u\right)}^{2}+{\left(R_{c}+r_{n}\right)}^{2}}\right).$$

The above is the expression of distance-to-frequency signal that does not produce Doppler ambiguity in the signal domain, so the problem of Doppler ambiguity can be completely solved after the above operation. After this, there is no Doppler ambiguity in the echo data. In this case, the data can be imaged by CS algorithm, wavenumber domain algorithm, polar coordinate algorithm and the like. In the following sections, we will discuss two SAR imaging algorithms suitable for sliding spotlight mode.

## 4. Convolution Backprojection Algorithm for Sliding Spotlight SAR

### 4.1. CBP Algorithm

**Figure 5:**
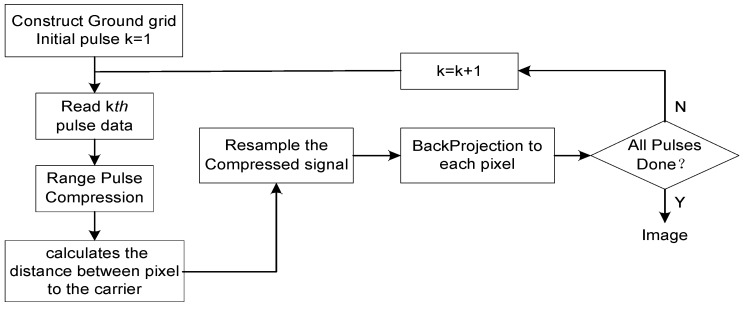
Flow chart of CBP algorithm.

The Convolution BackProjection (CBP) algorithm is a point-by-point imaging algorithm, which is a point-to-point image reconstruction process. The CBP algorithm can be artificially set according to the resolution requirements and the actual situation for different modes and different frequency bands. No matter how great the range migration is, the CBP algorithm can accumulate its energy for each point along its own migration curve [20]. The process is shown in Figure 5:

Step 1: Construct a ground pixel grid point

According to the resolution requirement, the pixel grid points of different pixel intervals are constructed in the ground imaging area, and the azimuth and distance coordinates of each pixel are recorded, to guarantee that the resolution in azimuth and range domain of the two-dimensional pixel unit are basically matched.

Step 2: Reverse-projection

(a) Range pulse compression

There is no need for multi-points accumulation after pulse compression, all oversampling points are left.

(b) Determination of the beam coverage of ground pixels

According to the corresponding azimuth of each pixel, the range coordinates, the antenna position and azimuth beam width of each pulse, it can be judged which of the pixels is within the coverage of the pulse beam and recorded.

The judgement is based on the two window functions in the echo expression of the ground target. The point target located in the two window functions can be covered by the pulse beam:

(19) $$w_{1}=\mathrm{rect}\left(\frac{\hat{t}-\frac{2R(t_{m};R_{B})}{c}}{T_{r}}\right).$$

(20) $$w_{2}=\mathrm{rect}\left(\frac{Avt_{m}-x}{X}\right)$$

(c) Pixel-by-pixel reverse-projection

Then we calculate the distance between each pixel in the imaging area and the corresponding antenna position of the pulse, and then the range data is interpolated according to the distance to obtain the different energy contribution of the pulse to the different pixels covered by the pulse. For the same pixel, the energy from different pulses of its contribution needs coherent accumulation.

In the time domain imaging algorithm such as CBP, the ground pixel are set artificially based on the resolution requirements and the actual situation, and the interval can be slightly smaller than the resolution generally, according to the desired image geometric direction.

### 4.2. Fast CBP Algorithm Based on Image Segmentation

The backprojection is a point to point image reconstruction process, which requires large amounts of interpolation operations resulting in huge computation [21]. Therefore, in this paper a fast implementation method based on Quadtree sub-image segmentation is discussed below.

In the pre-processing phase of the CBP algorithm, match filtering and motion compensation are needed according to the center of the scene, which are equivalent to the two-dimension dechirp processing of the original echo signal, eliminating the second order phase of the signal. The echo signal of single target after the two-dimensional dechirp is as follows [21]:

(21) $$S_{PB}\left(t,f_{\tau }\right)=\mathrm{rect}\left(\frac{t}{T_{a}}\right)\cdot \mathrm{rect}\left(\frac{f}{\gamma T_{r}}\right)\exp\left[j\frac{4\pi }{c}f_{\tau }\left(R_{a}-R_{p}\right)\right]\cdot \exp\left\{j\frac{4\pi }{c}f_{c}\left[R_{a}(t)-R_{p}(t)\right]\right\}.$$

The first exponential term is a nearly single frequency signal related to the distance from radar to the target, and the range profile can be obtained through Fourier transform with *f_τ_*. Suppose *w_r_* is the range scope of the scene, then the bandwidth in range domain after dechirp processing is:

(22) $$B_{r}=\frac{2w_{r}}{c}.$$

**Figure 6:**
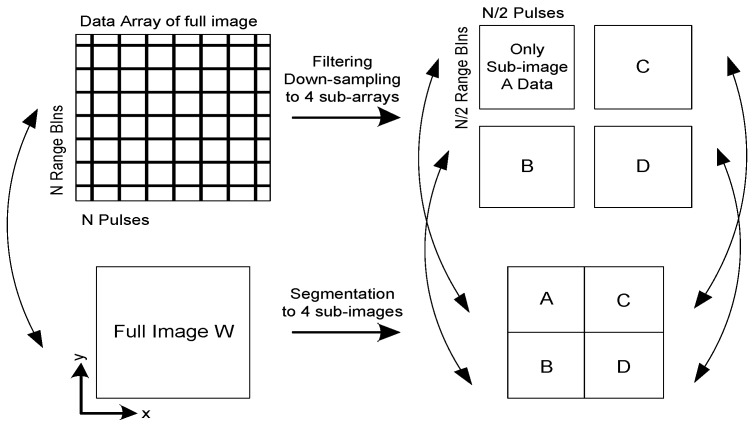
Schematic diagram of fast CBP algorithm based on image segmentation.

The second exponential term is also a nearly single frequency signal related to the azimuth position of the target, and the azimuth profile can be obtained through Fourier transform with *t*. Suppose *w_a_* is the azimuth scope of the scene, then the bandwidth in azimuth domain after dechirp processing is [22]:

(23) $$B_{a}=\frac{2w_{a}v}{\lambda_{c}R_{ac}}.$$

where *λ_c_* is the wavelength, *R_ac_* is the distance between the center point of the aperture and the center of the scene. In the CBP algorithm, if the scene decreases, the bandwidth in azimuth and range domain corresponding to the phase history will be reduced. Accordingly, the sampling rate can be reduced while increasing the sampling interval in azimuth and range domain to reduce the computation. Therefore, a CBP algorithm based on sub-image processing is used below. The schematic diagram is shown in Figure 6, and the specific process includes the following steps [23]:

Step 1: Sub-image segmentation

The segmentation of sub-image is based on the quadrant, and the whole image is divided into four sub-images according to the quadrant. The pixels are evenly distributed in the range and azimuth domain. For an image that includes *N × N* pixels, corresponding to Figure 6, each sub-image should contain *N*/2 × *N*/2 pixels. The scene is reduced to half of the original in both range and azimuth domain, so the sampling rate of data in range and azimuth domain can also be reduced to half in processing.

Step 2: Filtering the original phase history, then down-sample in the spatial frequency domain.

The filtering should be based on the range and the center point of each sub-image. The original phase history in the full scene is considered:

(24) $$S_{W}\left(t,f_{\tau }\right)=\mathrm{rect}\left(\frac{t}{T_{a}}\right)\cdot \iint_{(x,y)\in W}g(x,y)\cdot \mathrm{rect}\left(\frac{f_{\tau }}{B_{r}}\right)\cdot \exp\left[j\frac{4\pi }{c}(f_{c}+f_{\tau })\left(R_{a}-R_{t}\right)\right]\mathrm{dxdy}.$$

where *W* represents the ground illuminated area, and *g*(*x*, *y*) represents the reflectivity of point target with the coordinate of (*x*, *y*). The original phase history array size is *N × N*, and the sampling intervals in (*t*, *f_τ_*) domain are *T_0_* and *F_s_/N*. So the discrete values of *t* and *f_τ_* are:

(25) $$t=-\frac{N}{2}\cdot T_{0}+m\cdot T\;\;m=0,1,2,\ldots \ldots ,N-1$$

(26) $$f_{\tau }=-\frac{F_{s}}{2}+\frac{n\cdot F_{s}}{N}\;\;\;n=0,1,2,\ldots \ldots ,N-1$$

A basic image can be obtained from this data array through the basic linear RD algorithm [23]. Except the center of the scene, the other points could have some defocus. In order to avoid energy leakage caused by defocusing, motion re-compensation are needed, and then linear RD algorithm and low-pass filtering can be applied. In summary, the fast filtering process includes:

(a) Motion re-compensation to the center of the each sub-image.

For each sub-image, the motion compensation function is constructed based on the the central point in the sub-image, and the echo data is compensated by pulse by pulse. Taking sub-image A as an example, the phase compensation factor is:

(27) $$\phi_{sA}\left(t,f_{\tau }\right)=\exp\left[j\frac{4\pi }{c}f_{\tau }\left(R_{sA}-R_{a}\right)\right]\cdot \exp\left[j\frac{4\pi }{c}f_{c}\left(R_{sA}-R_{a}\right)\right].$$

where *R_sA_ = R_sA_(t)* is the instantaneous distance between the phase center of the antenna and the center of sub-image A, and it can be described in coordinates:

(28) $$R_{sA}=\sqrt{（x_{a}-x_{sA}{)}^{2}+（y_{a}-y_{sA}{)}^{2}+（z_{a}-z_{sA}{)}^{2}}.$$

The new phase history is obtained after phase compensation:

(29) $$S_{WcA}\left(t,f_{\tau }\right)=\mathrm{rect}\left(\frac{t}{T_{a}}\right)\cdot \iint_{(x,y)\in W}g(x,y)\cdot \mathrm{rect}\left(\frac{f_{\tau }}{B_{r}}\right)\cdot \exp\left[j\frac{4\pi }{c}(f_{c}+f_{\tau })\left(R_{sA}-R_{t}\right)\right]\mathrm{dxdy}.$$

(b) Two dimensional imaging and window interception.

At this time an image of the full scene can still be obtained by two-dimensional FFT, but now the center of the scene has been transferred to the center of each sub-image. Then it is convenient to extracting the sub-image data from the central part of the large two-dimensional data array of the full image according to the subscripts. The array size after interception is *N*/2 × *N*/2.

(c) Returning to the phase history domain.

The scene of this sub-image is reduced by half in both range and azimuth, so the sampling rate can be also reduced by half in both range and azimuth domain. After returning the image to the space-frequency domain through IFFT, the amount of data is reduced to the 1/4 of original, only containing the information of sub-image A. The signal returning to data domain is:

(30) $$S_{WcA}\left(t,f_{\tau }\right)=\mathrm{rect}\left(\frac{t}{T_{a}}\right)\cdot \iint_{(x,y)\in A}g(x,y)\cdot \mathrm{rect}\left(\frac{f_{\tau }}{B_{r}}\right)\cdot \exp\left[j\frac{4\pi }{c}(f_{c}+f_{\tau })\left(R_{sA}-R_{t}\right)\right]\mathrm{dxdy}.$$

Here the sampling points is half of the original, and the sampling interval is doubled. So the sampling intervals in (*t*, *f_τ_*) domain are 2*T*_0_ and 2*F_s_/N*. The discrete values of *t* and *f_τ_* are:

(31) $$t=-\frac{N}{2}\cdot T_{0}+2m\cdot T\;\;\;m=0,1,2,\ldots \ldots ,\frac{N}{2}-1.$$

(32) $$f_{\tau }=-\frac{F_{s}}{2}+\frac{n\cdot F_{s}}{N/2}\;\;\;n=0,1,2,\ldots \ldots ,\frac{N}{2}-1$$

**Figure 7:**
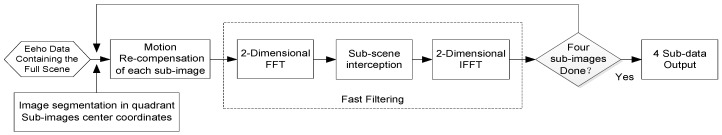
Flow diagram of fast filtering.

The flow diagram of fast filtering is shown in Figure 7.

Step 3: Backprojection.

Still taking sub-image A as an example, *S_sA_*(*t*, *f_τ_*) is the down-sample signal *P_θ_A_*(*U*) in frequency domain containing only information of sub-image A, so the reconstruction formula is changed into the following:

(33) $${\bar{g}}_{A}(x,y)=\int_{-\theta_{m}/2}^{\theta_{m}/2}\int_{U_{1}}^{U_{2}}P_{\theta \_A}(U)\cdot \exp\left(-jUR_{\Delta A}\right)\left|U\right|\mathrm{dUd}\theta .$$

The backprojection process is still realized by interpolation and summation. The pixel numbers of each sub-image is *N*/2 × *N*/2, and the pulse numbers for backprojection is also reduced to *N*/2, so the number of interpolation for each sub-image is *N*^3^/8 and total is *N*^3^/2 for all of the four sub-images, only half comparing to the normal process.

Step 4: Sub-images mosaic.

The full image is obtained by sub-images mosaic according to the original segmentation rules.

### 4.3. Further Improvement with Recursive Segmentation

The improvement of computation reduction is limited by only one level image segmentation so it is necessary to recursively segment the image further to reduce the sample rate and computation. As discussed below, it mainly includes the following two parts of recursive decomposition. Part one is to segment the image recursively. First the full image is decomposed into four sub-images by level-1 segmentation, and then each sub-image is decomposed into four smaller sub-images by level-2 segmentation again.

**Figure 8:**
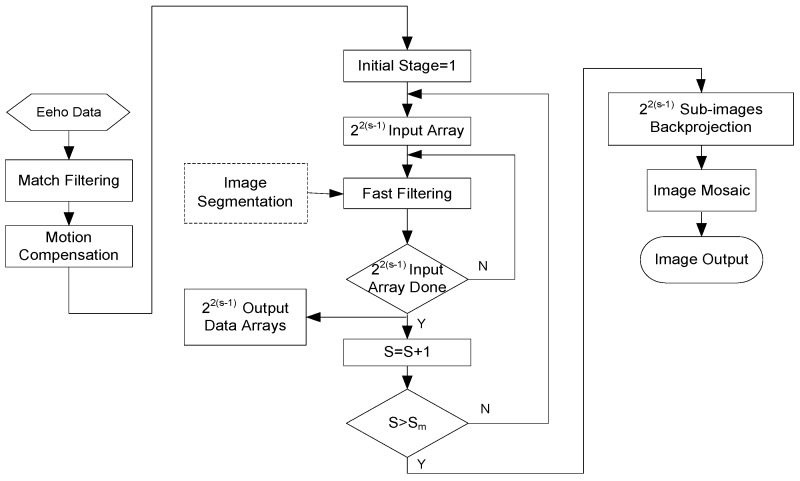
Flow chart of fast realization based on Quadtree sub-image recursive segmentation.

Part two is to segment the echo data recursively, including filtering and down-sampling. The original data is filtered according to the input sub-image parameters to ensure that the filtered data only contains the information of each sub-image. The flow diagram of fast CBP implementation is shown in Figure 8.

### 4.4. Simulation Results

**Figure 9:**
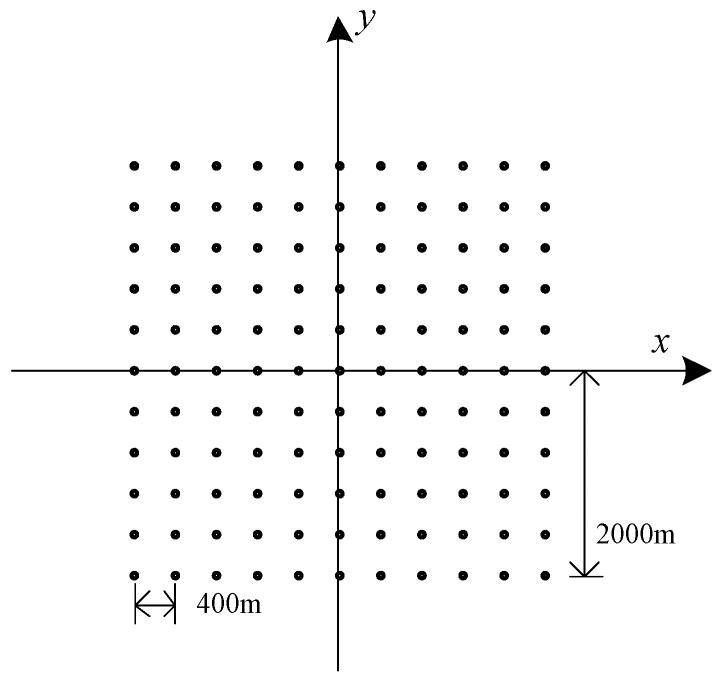
Simulation point targets distribution.

**Figure 10:**
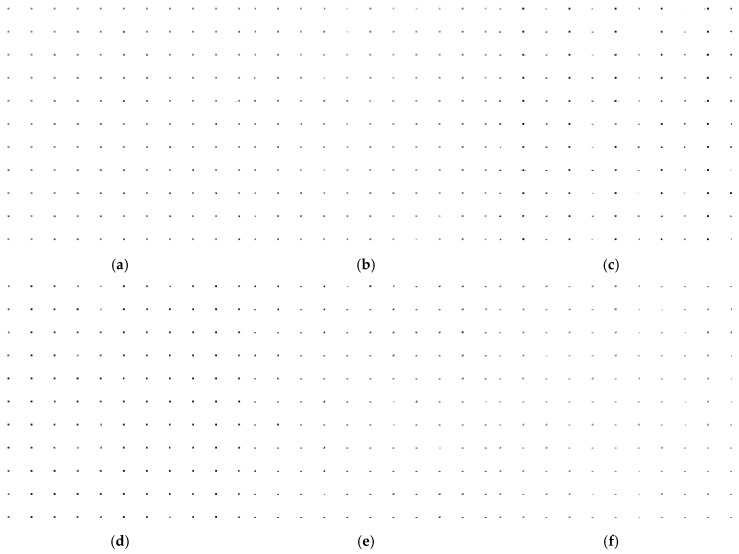
Imaging simulation result. (**a**) Divided to 128 × 128 points; (**b**) Divided to 64 × 64 points; (**c**) Divided to 32 × 32 points; (**d**) Divided to 16 × 16 points; (**e**) Divided to 8 × 8 points; (**f**) Divided to 4 × 4 points.

The simulation parameters are shown in Table 2, and the distribution maps of 121 simulation points are given in Figure 9. The space between each points is 400 m, and the imaging area is 4000 m × 4000 m. The radar echo signal after matching filter and motion compensation is a two dimensional matrix of 4096 × 4096 points. The result of fast imaging simulation is shown in Figure 10 with different segmentation levels.

The simulation results are shown in Figure 10, and the error positions of point targets in each image are estimated in Table 3.

**Figure 11:**
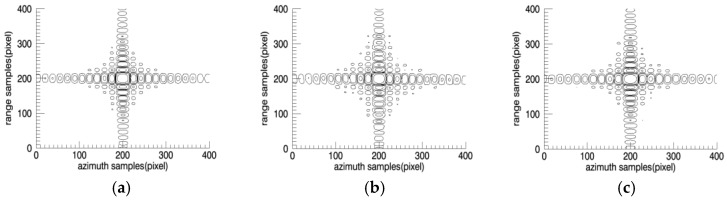
(**a**) Conventional CBP; (**b**) Divided to 32 × 32 points; (**c**) Divided to 16 × 16 points.

**Figure 12:**
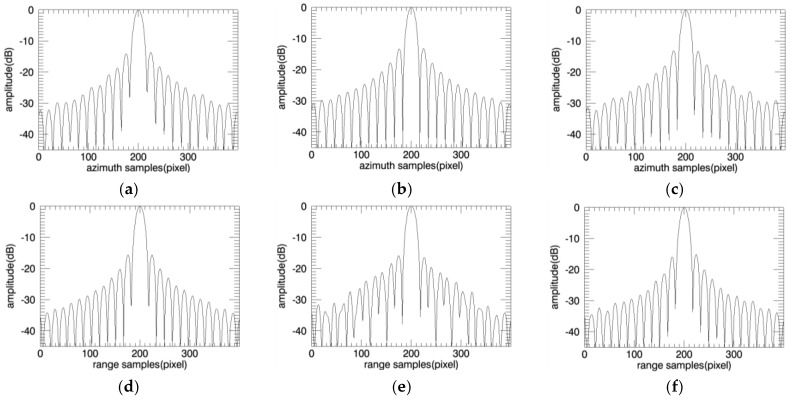
Imaging simulation result. Azimuth profile (**a**) Conventional CBP; (**b**) Divided to 32 × 32 points; (**c**) Divided to 16 × 16 points; Range profile (**d**) Conventional CBP; (**e**) Divided to 32 × 32 points; (**f**) Divided to 16 × 16 points.

The target response profiles are shown in Figure 11 and Figure 12. 

Evaluation results of point targets simulation are shown in Table 4.

From the above, it can be seen that all the images are well focused in each level. But each segmentation level is equivalent to re-sampling and cumulative error is inevitable, so the number of segmentation levels is a compromise between image quality and computation.

## 5. PFA Algorithm for Sliding Spotlight SAR

### 5.1. PFA Algorithm Based on Image Segmentation

The Polar Format Algorithm is a classical spotlight SAR imaging algorithm [24]. The algorithm uses the polar coordinate scheme to store the data, and effectively solves the problem of the cross-resolution unit moving away from the central scattering point of the imaging area. Since the PFA uses the assumption of the plane wavefront, and the actual wavefront is curved, the error introduced mainly appears as the primary and secondary spatial phase error of the spatial frequency, which corresponds to the geometric distortion and defocusing of image and limits the effective imaging scene size of PFA algorithm [25].

A novel PFA algorithm based on image quadtree partition of image is studied below, which shares the same key idea of the quadtree CBP algorithm and mainly includes three steps:

Step 1: Sub-image segmentation.

**Figure 13:**
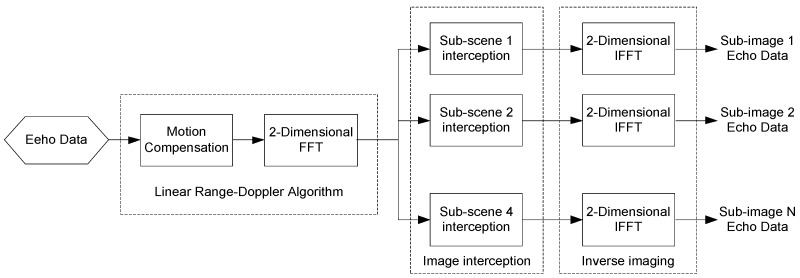
Diagram of pre-filtering process.

The basic LRD algorithm without precise focusing is used to get a blurred SAR image from the whole scene, and then based on the idea of digital spotlight, the echo data can be filtered and segmented into several sub-images through the quadtree method, which is shown in Figure 13.

Step 2: Motion compensation and PFA sub-imaging.

Remove the echo data of each sub-image to the center of each sub-scene, and use PFA algorithm for precise focusing. Taking the sub-scene center as the reference center, the second-order motion compensation for the data of each sub-image is given, and the reference function of compensation is:

(34) $${\bar{S}}_{n}^{}\left(t,f_{\tau }\right)=\exp\left\{j\frac{4\pi \left(f_{c}+f_{\tau }\right)}{c}\left[r_{n,o}^{}\left(t\right)-r_{0}\left(t\right)\right]\right\},\;n=1,2,\cdots ,\;N.$$

where *n* represents the *n*th sub-beam, *N* represents the number of sub-images, *r_n_*_,*o*_*(t)* represents the instantaneous distance from the satellite to the center of the *n*th sub-scene. The data of each sub-image after the motion compensation can be written as:

(35) $$S_{n}^{}\left(t,f_{\tau }\right)=\sum_{p\in {Ι}_{n}}^{}\exp\left\{j\frac{4\pi \left(f_{c}+f_{\tau }\right)}{c}\left[r_{n,o}^{}\left(t\right)-r_{p}\left(t\right)\right]\right\}\;.$$

Through the second-order motion compensation above, the motion error of the center of each sub-beam scene is accurately compensated. Although the compensation is invariant in the each sub-beam, as long as the sub-beam is designed narrow enough to ensure that the sub-scene is within the effective imaging scene radius of the PFA, the residual error of the non-center point can be completely negligible.

Step 3: Mosaic of sub-images.

The full scene free focus large graph can be obtained by mosaicking each sub-image. In the PFA algorithm based on sub-images, it is not necessary to a divide the full image into very small sub-images like in the CBP algorithm. The main purpose of fast CBP is to reduce the amount of interpolation in the backprojection process to reduce the computation, so the sub-images must be small enough to achieve this purpose effectively. The PFA imaging algorithm itself is very simple and efficient. Therefore, the level of sub-image segmentation can be reduced, as long as each sub-image is within the range of the effective focusing scene.

Under certain parameter conditions, the effective imaging scene radius of PFA is determined by the following formula [23]:

(36) $$r_{0}\leq \frac{2\rho_{a}}{K_{a}}\sqrt{\frac{R_{ac}}{\lambda_{c}}}.$$

**Figure 14:**
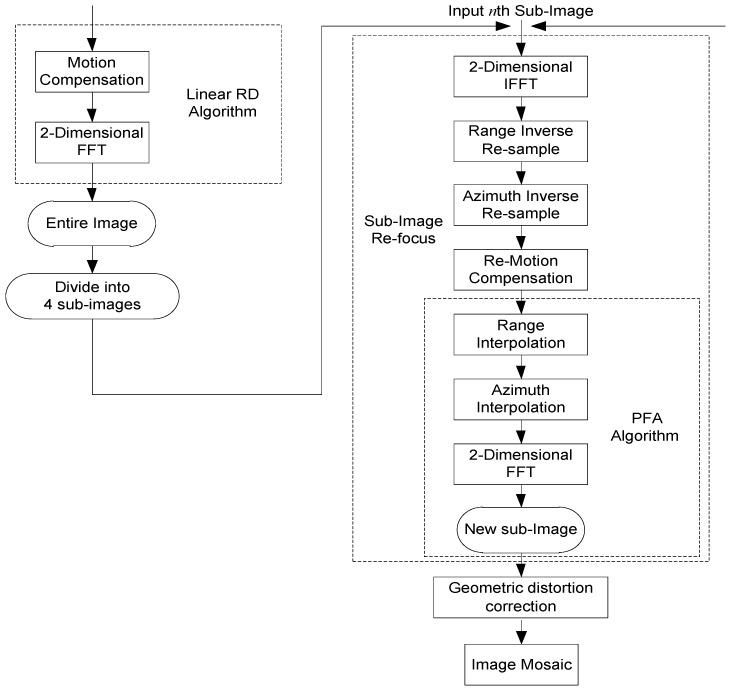
Flowchart of PFA algorithm based on sub-image segmentation.

According to the parameters of GF-3 SAR, it only needs Quadtree segmentation for only one time to satisfy the sub-image without defocus. Similar to the CBP algorithm, the flowchart of PFA algorithm based on Quadtree sub-image segmentation is shown in Figure 14.

### 5.2. Simulation Results

**Figure 15:**
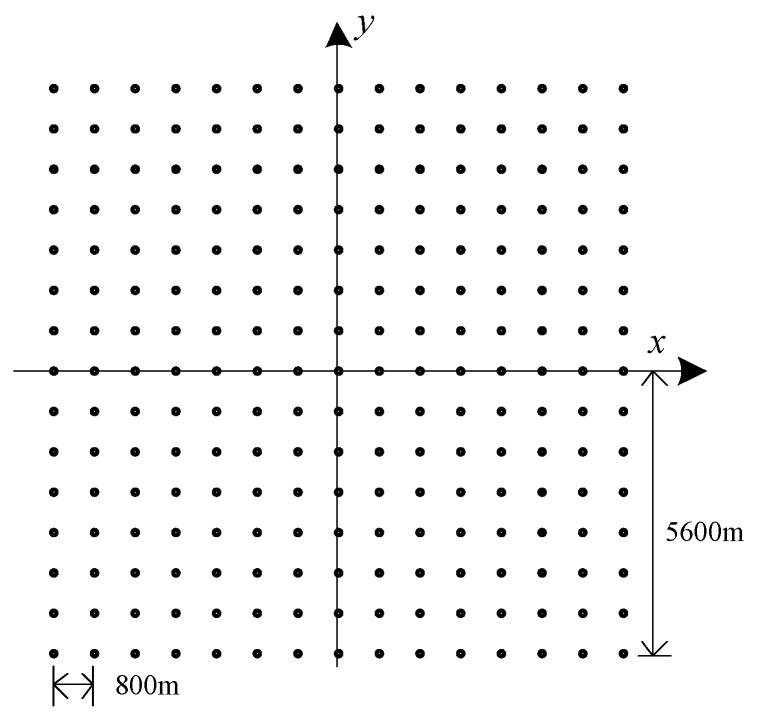
Simulation point targets distribution.

The simulation parameters are same with Table 2, and the distribution of 225 simulation points are given in Figure 15. The space between each points is 800 m, and the imaging area is 11,200 m × 11,200 m, which is far beyond the effective imaging scene size. According to the relationship between the actual scene range and the effective scene range, we can find that only one level segmentation of 4 sub-images can be controlled within the effective imaging scene radius.

**Figure 16:**
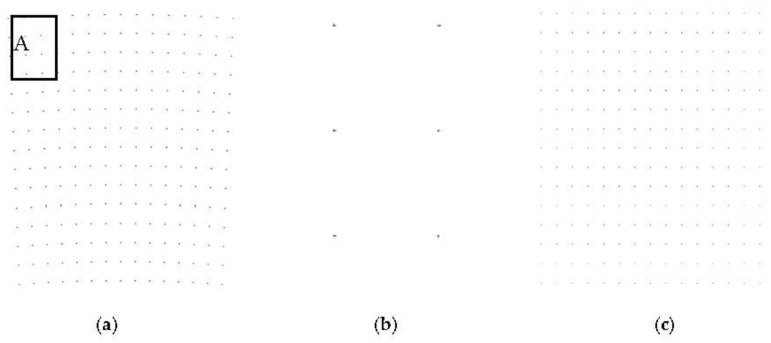
(**a**) Result of PFA; (**b**) Local sub-image; (**c**) Full image after mosaic.

The echo signal after matching filter and motion compensation is a two dimensional matrix of 16,384 × 16,384 points. Image obtained by PFA is shown in Figure 16a, while the corresponding imaging range is beyond the actual scene, it can be seen that the image has obvious geometric distortion, and error position of the point target is max to 15 and 16 m in range and azimuth domain compared to the original point target distribution in region A.

**Figure 17:**
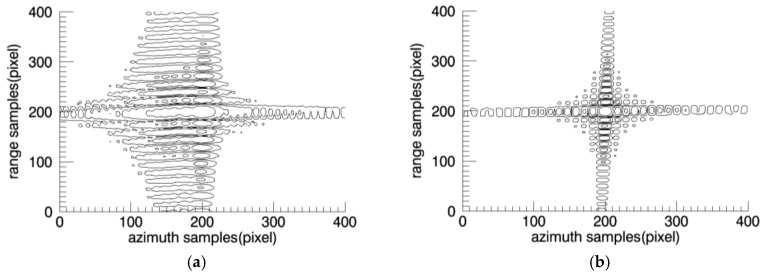
Two dimensional characteristic of point target. (**a**) PFA; (**b**) PFA based on sub-images.

**Figure 18:**
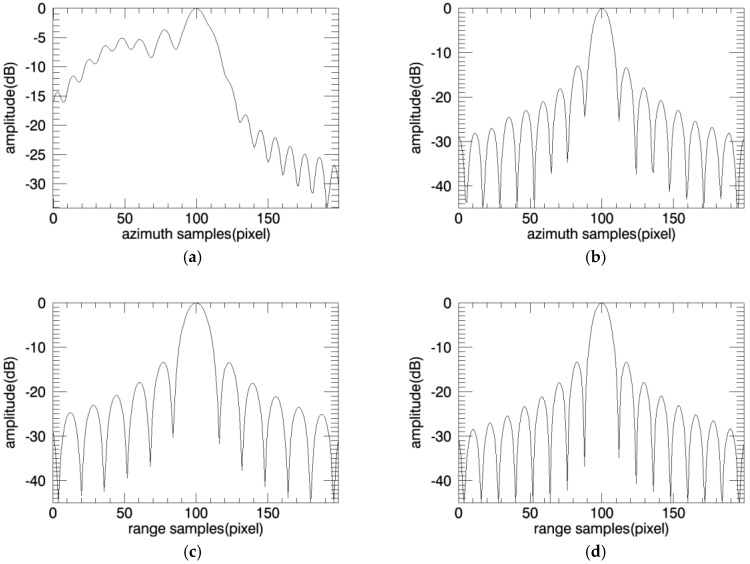
Comparison of point target profile. (**a**) Azimuth profile of PFA; (**b**) Azimuth profile of PFA based on sub-images; (**c**) Range profile of PFA; (**d**) Range profile of PFA based on sub-images.

The imaging result of one sub-image is shown in Figure 16b, corresponding to region A. It can be seen from the figure, the distortion has been preliminarily corrected. The full image after mosaic is shown in Figure 16c, and the geometric distortion has been all corrected, well focus and with no error position. The target response characteristics are shown in Figure 17 and Figure 18.

Evaluation results of point targets simulation are shown in Table 5.

## 6. Measured Data Results

Furthermore, we validate the proposed PFA algorithm by the measured data of an airborne SAR. The slant range of the data is 25 km, and the theoretical resolution is 0.15 m × 0.15 m. Under these parameters, the effective imaging scene radius of PFA is about 300 m, and the corresponding imaging area is about 3600 m and 1100 m in the azimuth and range domain respectively, which is far beyond the effective imaging scene of PFA. Therefore, it is necessary to divide the whole image into small sub-images and re-focus in order to improve the image quality.

After LRD processing without precise focusing, a basic image with a two dimensional matrix of 8192 × 24,000 points is obtained. According to the relationship between the effective radius of PFA imaging and the whole scene size, the whole image is divided into 11 × 16 = 176 sub-images. Each sub-image contains 1024 × 2048 pixels with overlapping 256 × 512 pixels.

**Figure 19:**
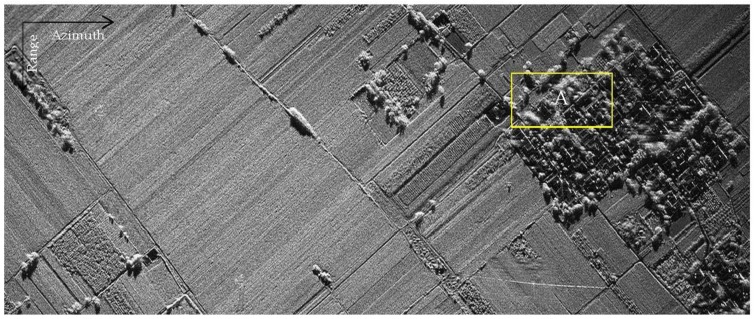
Measured data results of PFA based on sub-images.

**Figure 20:**
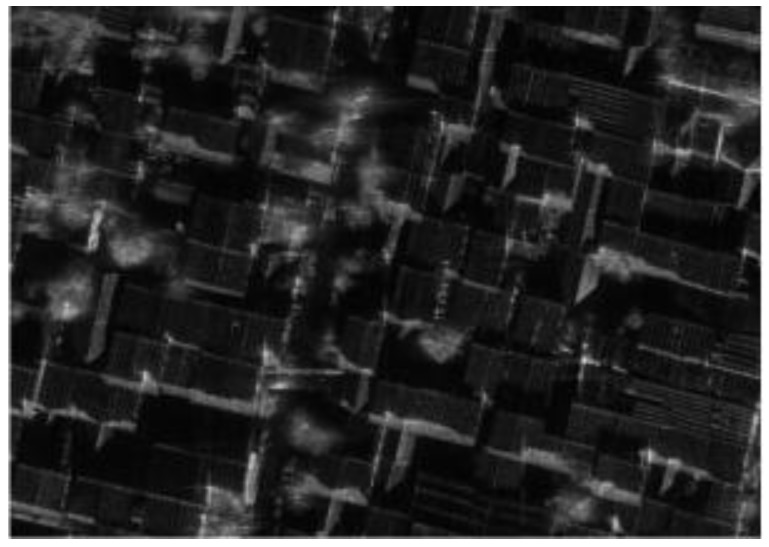
Detail of the local area.

**Figure 21:**
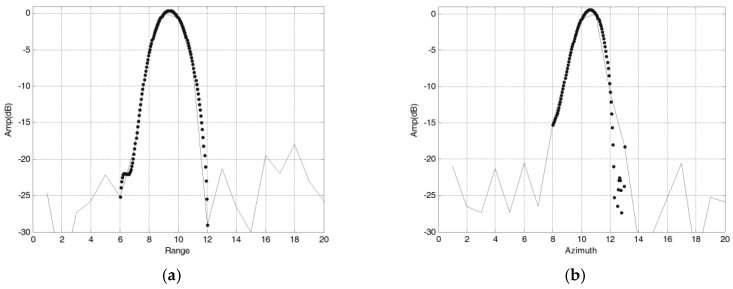
Point target profile. (**a**) Range profile; (**b**) Azimuth profile.

Measured data results of modified PFA based on sub-images are shown in Figure 19, and a local area in the image is shown in Figure 20 specifically. It can be seen that the modified PFA algorithm can significantly improve the focusing effect of the image. Figure 21 is the profile of point target in the image, and Hamming window is used in RAW data process on order to reduce the sidelobe levels.

Evaluation results of point targets in the SAR image are shown in Table 6.

## 7. Conclusions

In this paper, the key techniques in spaceborne sliding spotlight SAR are analyzed, including the imaging geometric parameters, the method of equivalent velocity acquisition and ambiguity elimination. The mechanism of the CBP algorithm and PFA algorithm for SAR imaging are studied, and the process of the fast application in spaceborne sliding spotlight SAR is analyzed in detail.

The large amount of computation is the main problem that restricts the application of CBP. In order to reduce the computation, a fast implementation method based on Quadtree sub-images is studied in this paper. The azimuth filtering and down sampling based on sub-image interception and FFT method for fast filtering is studied to avoid interpolation process, and through the method of Quadtree recursive decomposition, the processing efficiency of CBP can be greatly improved and the image quality can be ensured.

PFA is also a classical algorithm for spotlight SAR. In order to expand the scope of PFA effective scenes for the sliding spotlight mode, based on the idea of sub-images, a modified PFA algorithm suitable for a large scene is proposed. As long as the sub-images are small enough, it is within the effective range of traditional PFA imaging scene restrictions. Finally, after geometric distortion correction, all sub-images are stitched to get the full image without defocus.

Based on the proposed algorithm, the sliding spotlight SAR imaging can be realized quickly according to the process, and the algorithms are validated by simulation and measured data to lay the foundation for future spaceborne applications.

## Figures and Tables

**Table 1:** Orbit simulation parameters.

Parameters	Discription	Value
*A*/Km	Semimajor axis	7000
*e*	Eccentricity	0.001
*i*/*°*	Inclination	97.0
*Ω*/*°*	Ascending node	−0.0999845
*ω*/*°*	Argument of perigee	−0.0121851

**Table 2:** Simulation parameters.

Parameters	Value	Parameters	Value
Carrier Frequency	5.4 GHz	Wavelength	0.056 m
Signal bandwidth	240 MHz	Sampling frequency	300 MHz
Height of platform	750 Km	Pulse width	20 us
Incidence angle	30°	Size of sub-images	4 × 4~128 × 128
Azimuth size of antenna	7.5 m	Type of window	None
Range resolution	1.0 m	Azimuth resolution	1.2 m

**Table 3:** Error positions of the point targets.

Parameters	Mean Error (Pixel)	Standard Deviation (Pixel)
Divided to 128 × 128 points	0.22	0.06
Divided to 64 × 64 points	0.31	0.10
Divided to 32 × 32 points	0.37	0.12
Divided to 16 × 16 points	0.44	0.17
Divided to 8 × 8 points	0.49	0.21
Divided to 4 × 4 points	0.52	0.24

**Table 4:** Evaluation results of point targets simulation.

	Conventional CBP	Divided to 32 × 32 Points	Divided to 16 × 16 Points
Theoretical Range Resolution	1.00 m		
Simulation Range Resolution	1.05 m	1.06 m	1.09 m
Range PSLR	−13.71 dB	−13.55 dB	−13.23 dB
Theoretical Azimuth Resolution	1.19 m		
Simulation Azimuth Resolution	1.23 m	1.26 m	1.31 m
Azimuth PSLR	−13.51 dB	−13.15 dB	−13.03 dB

**Table 5:** Evaluation results of point targets simulation.

	Conventional PFA	PFA with Sub-Images
Theoretical Range Resolution	1.00 m	
Simulation Range Resolution	1.18 m	1.05 m
Range PSLR	−13.23 dB	−13.26 dB
Theoretical Azimuth Resolution	1.19 m	
Simulation Azimuth Resolution	−	1.22 m
Azimuth PSLR	−7.15 dB	−12.86 dB

**Table 6:** Evaluation results of point targets.

	Range Resolution	Range PSLR	Azimuth Resolution	Azimuth PSLR
Modified PFA	0.16 m	21.5 dB	0.18 m	20.9 dB
